# Supplementary material for: Deficiency of the lipid flippase ATP10A causes diet-induced dyslipidemia in female mice
Source: Sci Rep. 2024 Jan 3;14:343. doi: 10.1038/s41598-023-50360-5 (PMC10764864; doi:10.1038/s41598-023-50360-5)
Supplement: Supplementary file 1 — Supplementary Legends. [file 41598_2023_50360_MOESM1_ESM.docx]

Supplemental Table 1. ***Atp10A* deletion causes alterations to average daily food intake and energy balance during the light hours in female mice fed a HFD for 13 weeks.** The table indicates the parameters measured in the Sable System’s Promethion Indirect Calorimetry System where the mice were single-housed. The values are an average measurement from 5 days in the system with ad libitum feeding. P value by unpaired t-test (*10A^+/+^* n=7, *10A^-/-^* n=13), ns=nonsignificant.

Supplemental Table 2. ***Atp10A* deletion results in changes to the expression of genes involved in several biological pathways in visceral fat after HFD feeding.** Gene enrichment report for visceral fat mRNA gene expression data from *10A*^+/+^ and *10A*^-/-^ female mice (Figure 4C) created using the Kyoto Encyclopedia of Genes and Genomes (KEGG) database. FDR= false discovery rate, padj= p-value adjusted for multiple testing.

Supplemental Table 3. **Characteristics of mice used in this study.** The table includes information on sex, sample size, body mass after HFD feeding, age at time of sacrifice, experimental conditions before sacrifice, and statistical outlier testing.

Supplemental Figure 1. **Aligned sequence trace files of *Atp10A* exon 2 region from *WT* and *KO* mice.** Sequencing reactions were primed from the intron 5’ of exon 2. The trace from *Atp10A* WT mice has the expected sequence and the trace from KO mice shows the deletion junction that merges upstream and downstream introns with the removal of exon 2.

Supplemental Figure 2. ***Atp10A* deletion does not affect weight gain, body composition, or glucose homeostasis in female mice after 12 weeks on normal chow.** (a) Weight gain of *10A^+/+^* and *10A^-/-^* female mice over the course of 12 weeks on normal chow (4.5 kcal% fat, Ad lib feeding), (*10A^+/+^* n=4,*10A^-/-^* n=7). (b) Lean and (c) fat body mass was normalized to the combined sum of lean and fat mass to calculate % Lean and % Fat mass (*10A^+/+^* n=4, *10A^-/-^* n=4). (d) Body length of mice was measured after CO_2_ sacrifice (*10A^+/+^* n=4,*10A^-/-^* n=4). (e) Fasting blood glucose was measured a 5 hour fast, via a glucometer (*10A^+/+^* n=4,*10A^-/-^* n=4). (f) Fasting plasma insulin was measured after a 5 hour fast (*10A^+/+^* n=4,*10A^-/-^* n=4). P value by (a) 2-way ANOVA with Sidak’s multiple comparison or (b-f) unpaired t-test.

Supplemental Figure 3. **Maternally inherited** ***Atp10A* deletion leads to smaller bodies and**

**elevated fasting blood glucose in male mice on the 12th week on the HFD.** (a) Weight gain of heterozygous males and females inheriting the KO allele maternally (Maternal Inheritance (Inh.), *10A^+/-^* dam X *10A*^+/+^ sire) or paternally (Paternal Inh, *10A*^+/+^ dam x *10A^+/-^* sire) over the course of 12 weeks on a HFD (60 kcal% fat, Ad lib feeding) (**Males**: Maternal Inh.*10A^+/-^* n=7, Paternal Inh.*10A^+/-^* n=10; **Females**: Maternal Inh.*10A^+/-^* n=10, Paternal Inh.*10A^+/-^* n=6). (b) Lean and (c) fat body mass were normalized to the combined sum of lean and fat mass to calculate % Lean and % Fat mass (**Male**: Maternal Inh.*10A^+/-^* n=7, Paternal Inh.*10A^+/-^* n=10; **Female**: Maternal Inh.*10A^+/-^* n=10, Paternal Inh.*10A^+/-^* n=6). (d) Body length of male mice was measured after CO_2_ sacrifice, *P=0.0490. (Maternal Inh.*10A^+/-^* n=4, Paternal Inh.*10A^+/-^* n=9). (e) Fasting blood glucose was measured after a 5 hour fast, via a glucometer, **P=0.0081. (**Male**: Maternal Inh.*10A^+/-^* n=7, Paternal Inh.*10A^+/-^* n=10; **Female**: Maternal Inh.*10A^+/-^* n=10, Paternal Inh.*10A^+/-^* n=6). (f) Fasting plasma insulin was measured after a 5 hour fast (**Male**: Maternal Inh.*10A^+/-^* n=6, Paternal Inh.*10A^+/-^* n=10; **Female**: Maternal Inh.*10A^+/-^* n=5, Paternal Inh.*10A^+/-^* n=2). (a) P value by 2-way ANOVA with Sidak’s multiple comparison or (b-f) unpaired t-test.

Supplemental Figure 4. ***Atp10A* deficiency does not result in dyslipidemia in male mice after HFD feeding.** (a-c) Free fatty acids (FFA), cholesterol (chol), and triglycerides (TG) were measured in plasma from males after a 5 hour fast. P value by unpaired t-test. (a, *10A^+/+^* n=7, *10A^-/-^* n=6; b, *10A^+/+^* n=8, *10A^-/-^* n=6; c, *10A^+/+^* n=9, *10A^-/-^* n=7).

Supplemental Figure 5. ***Atp10A* deletion does not affect total amounts of liver cholesterol, TGs, PLs, or ceramides in female mice after 12 weeks of HFD.** Total (a) cholesterol, (b) cholesterol esters, (c) unesterified cholesterol, (d) TGs, (e) PLs, and (f) ceramides were measured from flash frozen livers via gas chromatography. Livers were collected after a 5 hour fast or after a 5 hour fast followed by an OGTT. P value by unpaired t-test (Total cholesterol: *10A^+/+^* n=16, *10A^-/-^* n=7; Cholesterol esters: *10A^+/+^* n=6,*10A^-/-^* n=6; Unesterified cholesterol: *10A^+/+^* n=6,*10A^-/-^* n=6; TG: *10A^+/+^* n=14, *10A^-/-^* n=7; PLs: *10A^+/+^* n=16, *10A^-/-^* n=7; Ceramides: *10A^+/+^* n=16, *10A^-/-^* n=7).

Supplemental Figure 6. ***Atp10A* deletion results in changes to total levels of two free fatty acid species and one cholesterol ester species in liver from female mice after 12 weeks of HFD.** The total amount of several different (a) FFA, (b) TG, (c) PL, (d) CE, and (e) ceramide species were measured from flash frozen livers via gas chromatography. Livers were collected after a 5 hour fast or after a 5 hour fast followed by an OGTT. P values by 2-way ANOVA with Sidak’s multiple comparisons test, ***P=0.0004, ****P=<0.0001.
