# Supplementary material for: Deficiency of the lipid flippase ATP10A causes diet-induced dyslipidemia in female mice
Source: Sci Rep. 2024 Jan 3;14:343. doi: 10.1038/s41598-023-50360-5 (PMC10764864; doi:10.1038/s41598-023-50360-5)
Supplement: Supplementary file 2 — Supplementary Figures. [file 41598_2023_50360_MOESM2_ESM.pdf]

Supplementary Materials

Deficiency of the lipid flippase ATP10A causes diet-induced dyslipidemia in female mice

Adriana C. Norris[1], Eugenia M. Yazlovitskaya[1], Lin Zhu[2], Bailey S. Rose[3,4,5,6,7], Jody C. May[3,4,5,6,7], Katherine N. Gibson-Corley[8], John A. McLean[3,4,5,6,7], John M. Stafford [2,9,10], Todd R. Graham[1]*

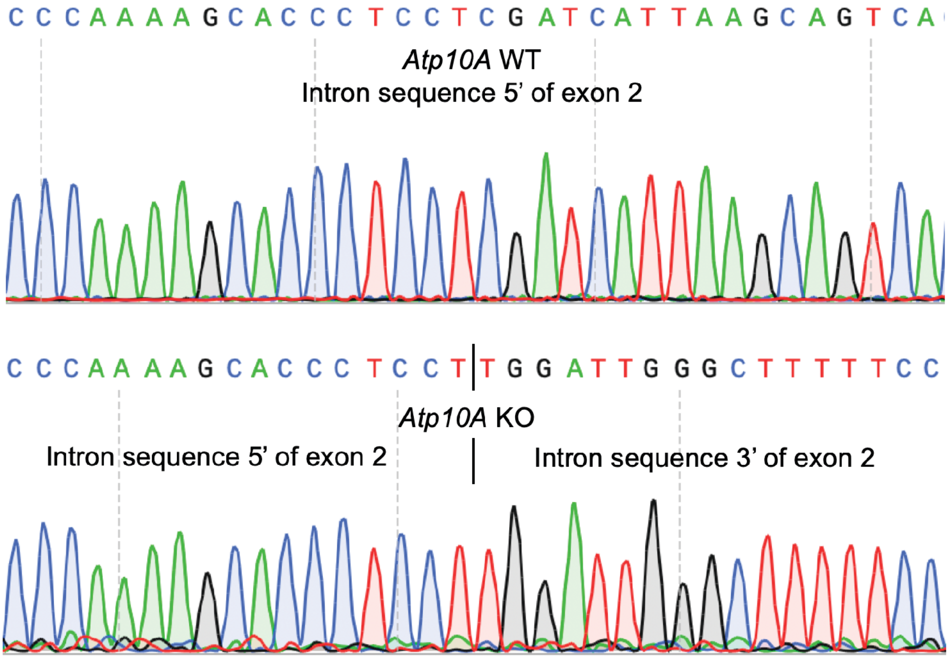

Supplemental Figure 1

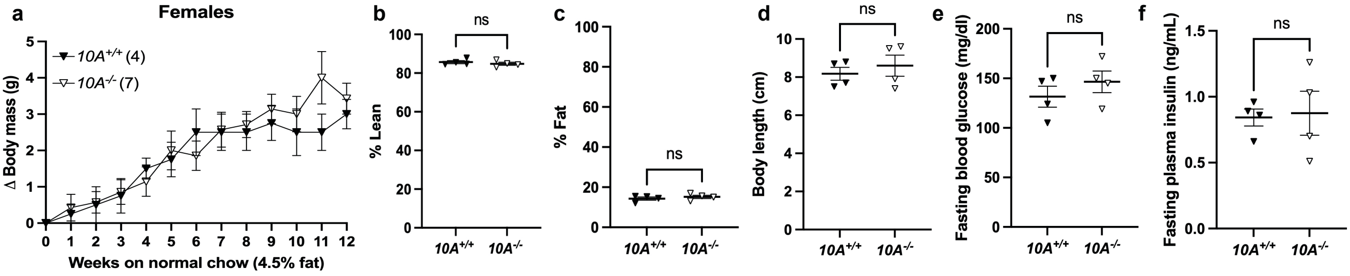

Supplemental Figure 2

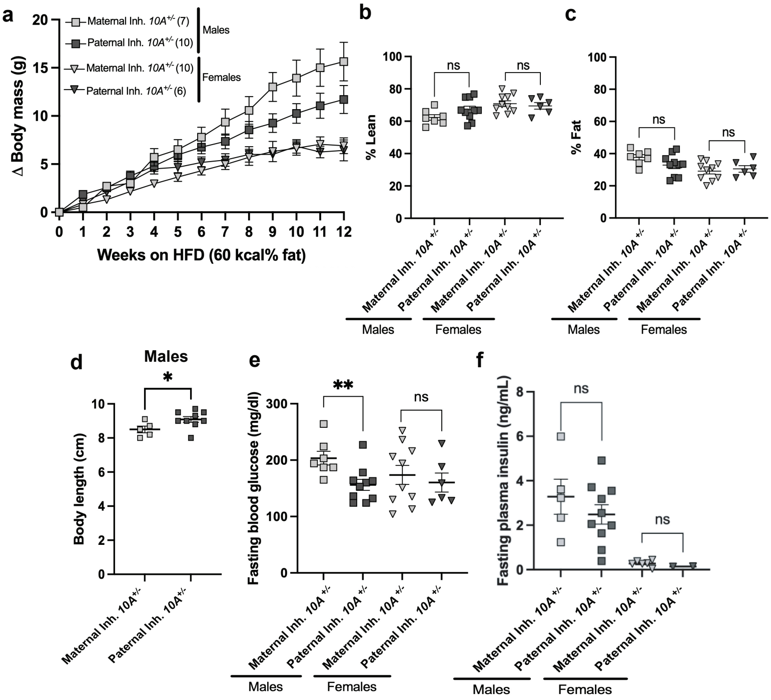

Supplemental Figure 3

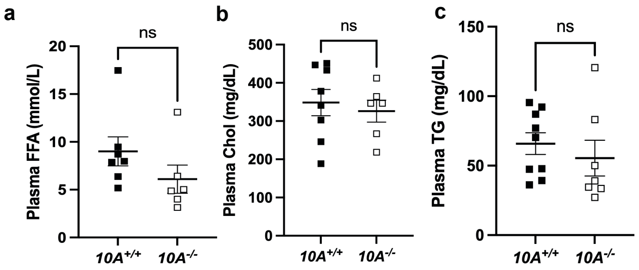

Supplemental Figure 4

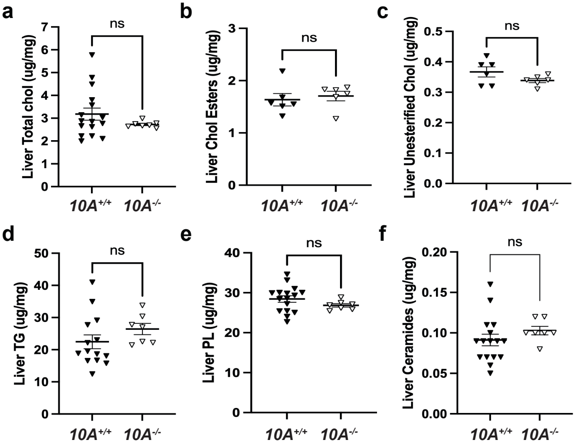

Supplemental Figure 5

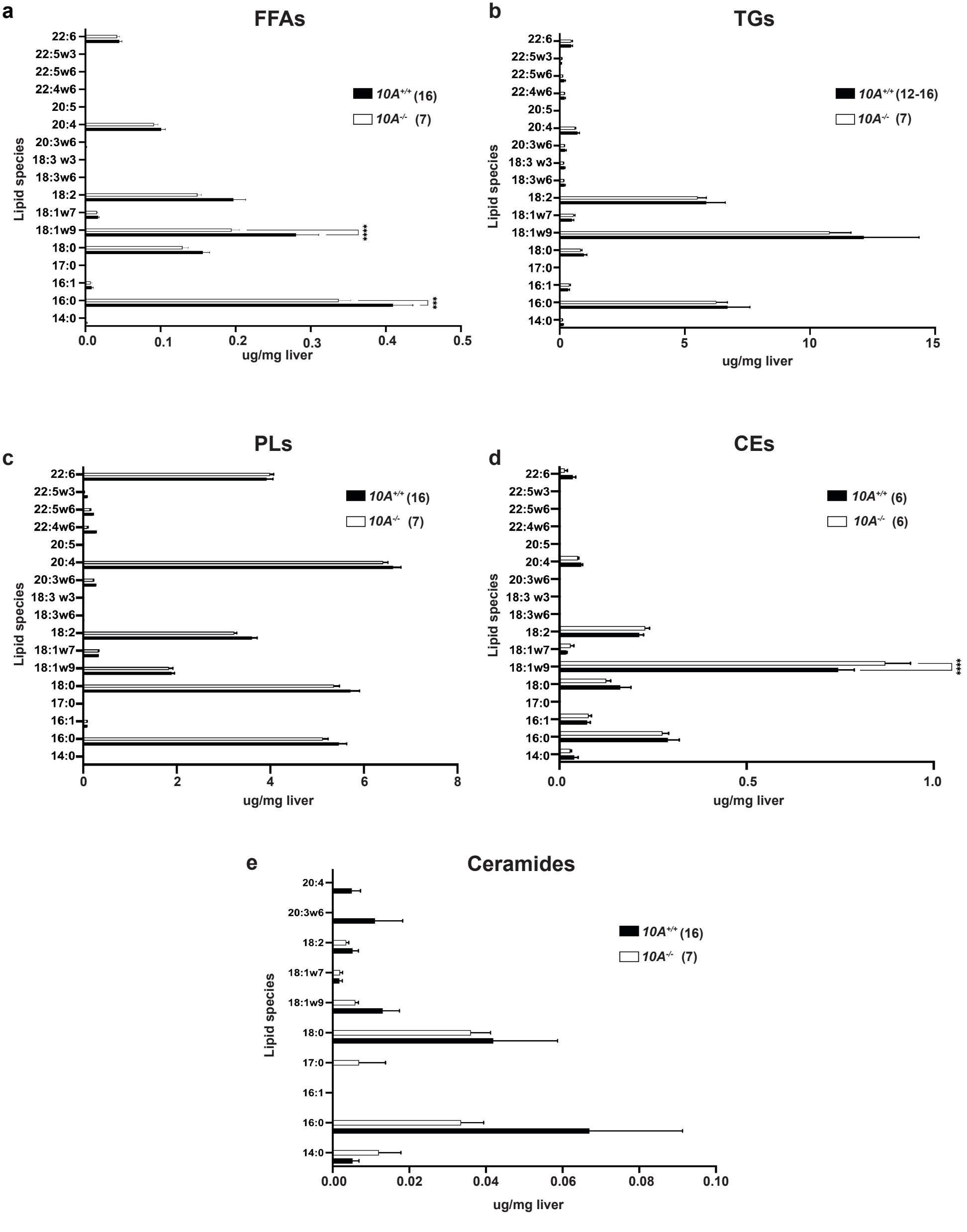

Supplemental Figure 6
