# Supplementary material for: Deficiency of the lipid flippase ATP10A causes diet-induced dyslipidemia in female mice
Source: Sci Rep. 2024 Jan 3;14:343. doi: 10.1038/s41598-023-50360-5 (PMC10764864; doi:10.1038/s41598-023-50360-5)
Supplement: Supplementary file 3 — Supplementary Figure 7. [file 41598_2023_50360_MOESM3_ESM.pptx]

## Slide 1
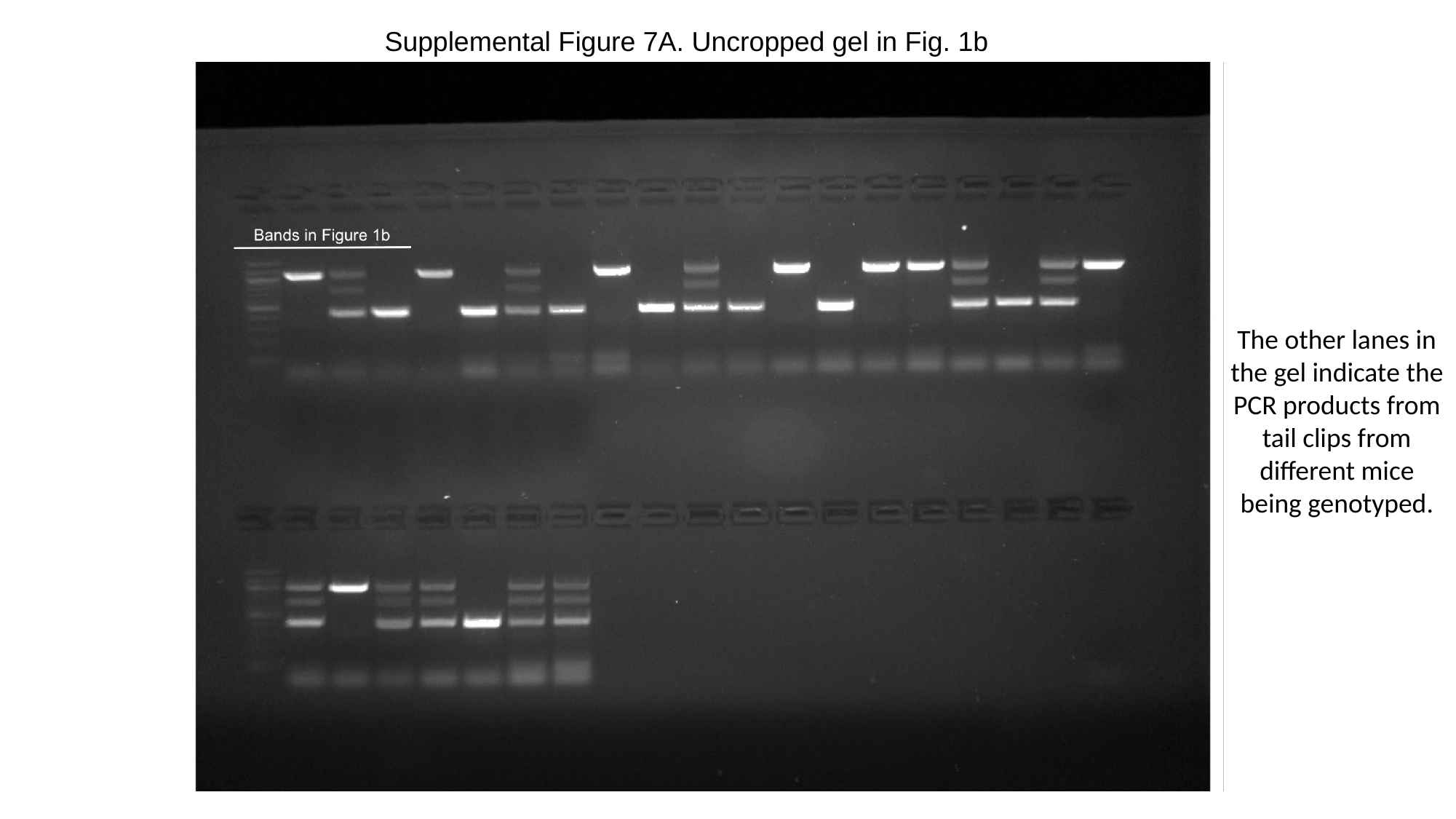

Supplemental Figure 7A. Uncropped gel in Fig. 1b
The other lanes in the gel indicate the PCR products from tail clips from different mice being genotyped.

## Slide 2
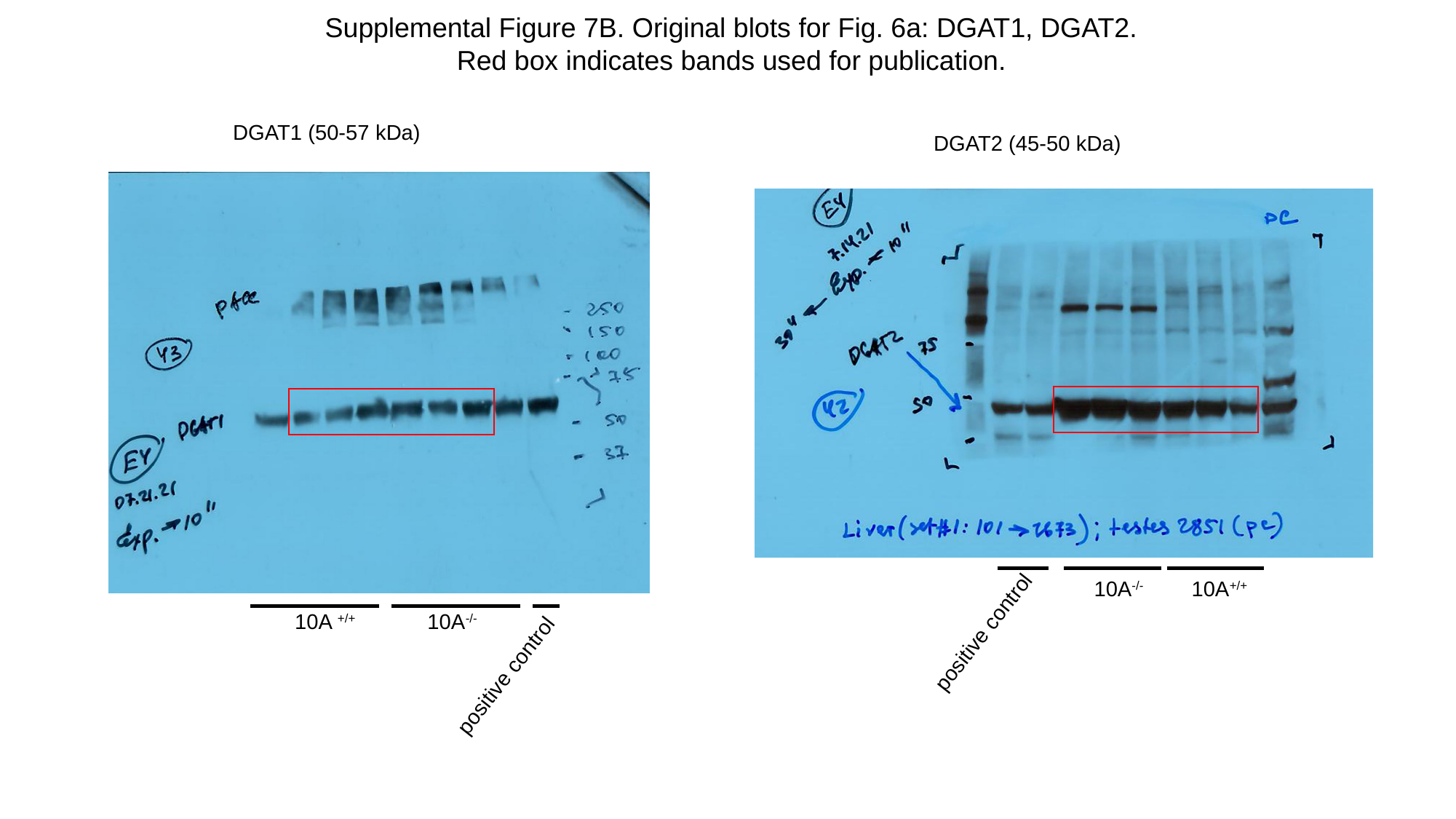

Supplemental Figure 7B. Original blots for Fig. 6a: DGAT1, DGAT2.
Red box indicates bands used for publication.
DGAT1 (50-57 kDa)
DGAT2 (45-50 kDa)
10A-/- 10A+/+
10A +/+ 10A-/-
positive control
positive control

## Slide 3
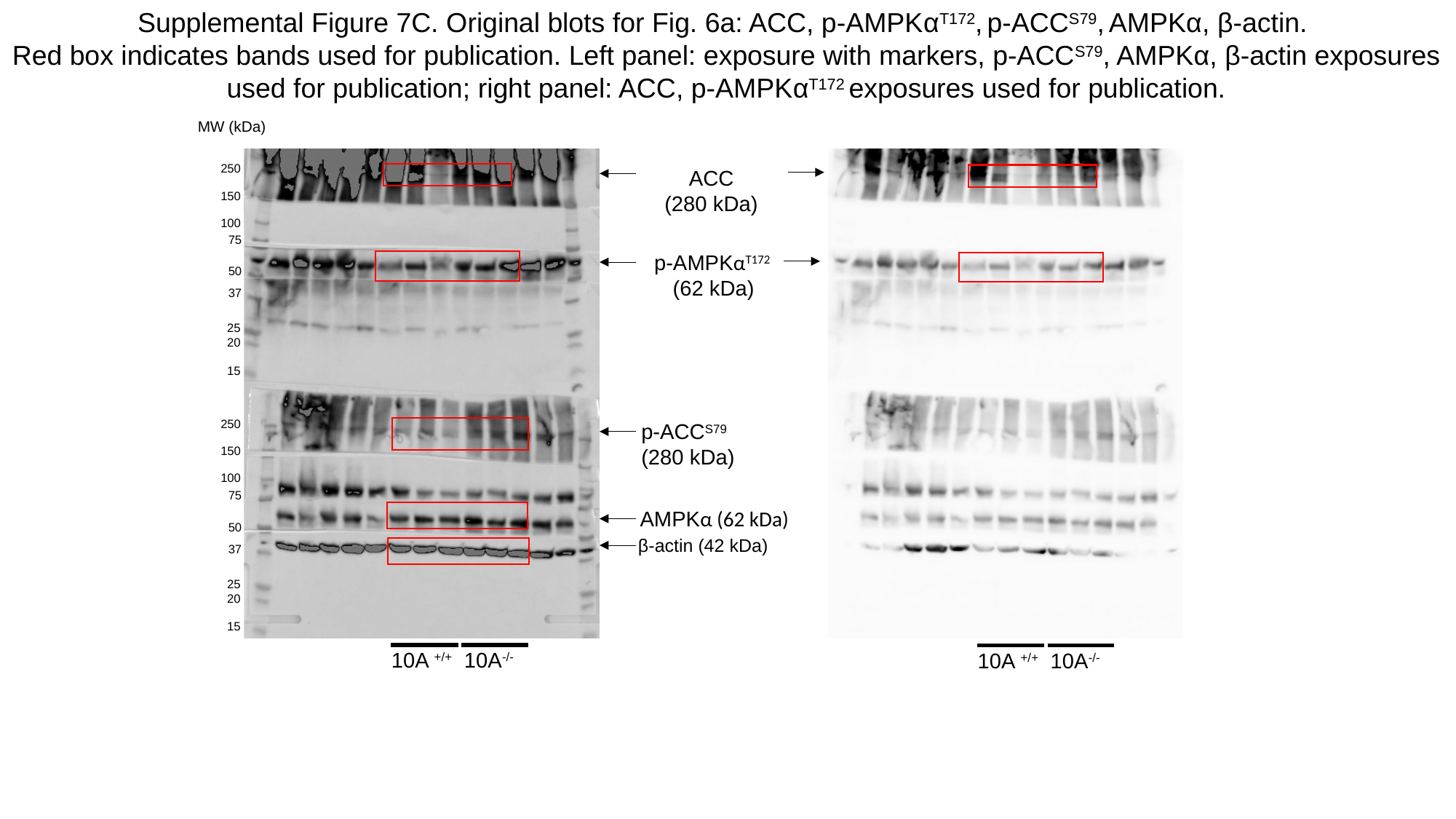

Supplemental Figure 7C. Original blots for Fig. 6a: ACC, p-AMPKαT172, p-ACCS79, AMPKα, β-actin.
Red box indicates bands used for publication. Left panel: exposure with markers, p-ACCS79, AMPKα, β-actin exposures used for publication; right panel: ACC, p-AMPKαT172 exposures used for publication.
MW (kDa)
250
ACC
(280 kDa)
150
100
75
p-AMPKαT172
(62 kDa)
50
37
25
20
15
250
p-ACCS79
(280 kDa)
150
100
75
AMPKα (62 kDa)
50
β-actin (42 kDa)
37
25
20
15
10A +/+ 10A-/-
10A +/+ 10A-/-

## Slide 4
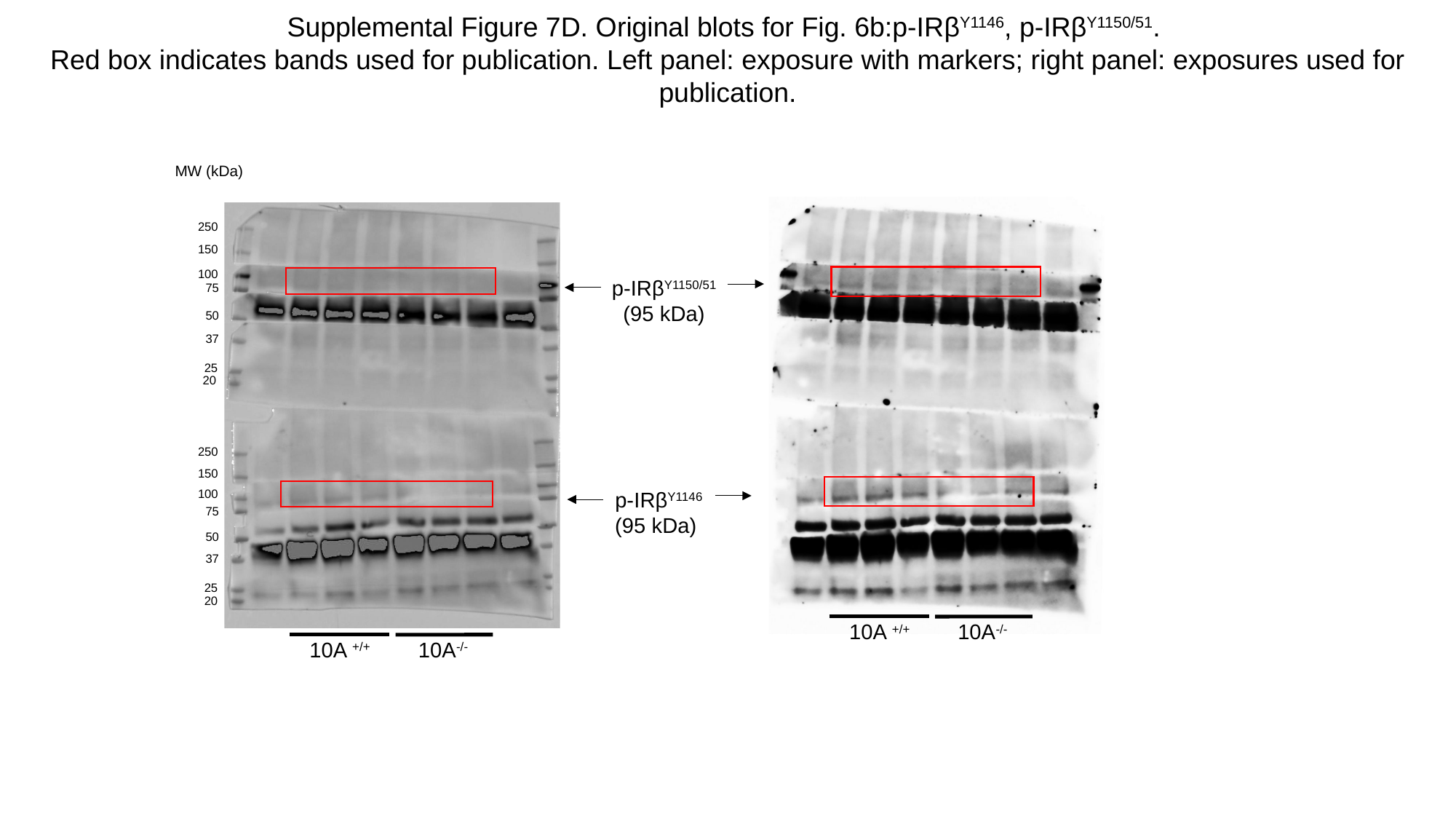

Supplemental Figure 7D. Original blots for Fig. 6b:p-IRβY1146, p-IRβY1150/51.
Red box indicates bands used for publication. Left panel: exposure with markers; right panel: exposures used for publication.
MW (kDa)
250
150
100
p-IRβY1150/51
(95 kDa)
75
50
37
25
20
250
150
100
p-IRβY1146
(95 kDa)
75
50
37
25
20
10A +/+ 10A-/-
10A +/+ 10A-/-

## Slide 5
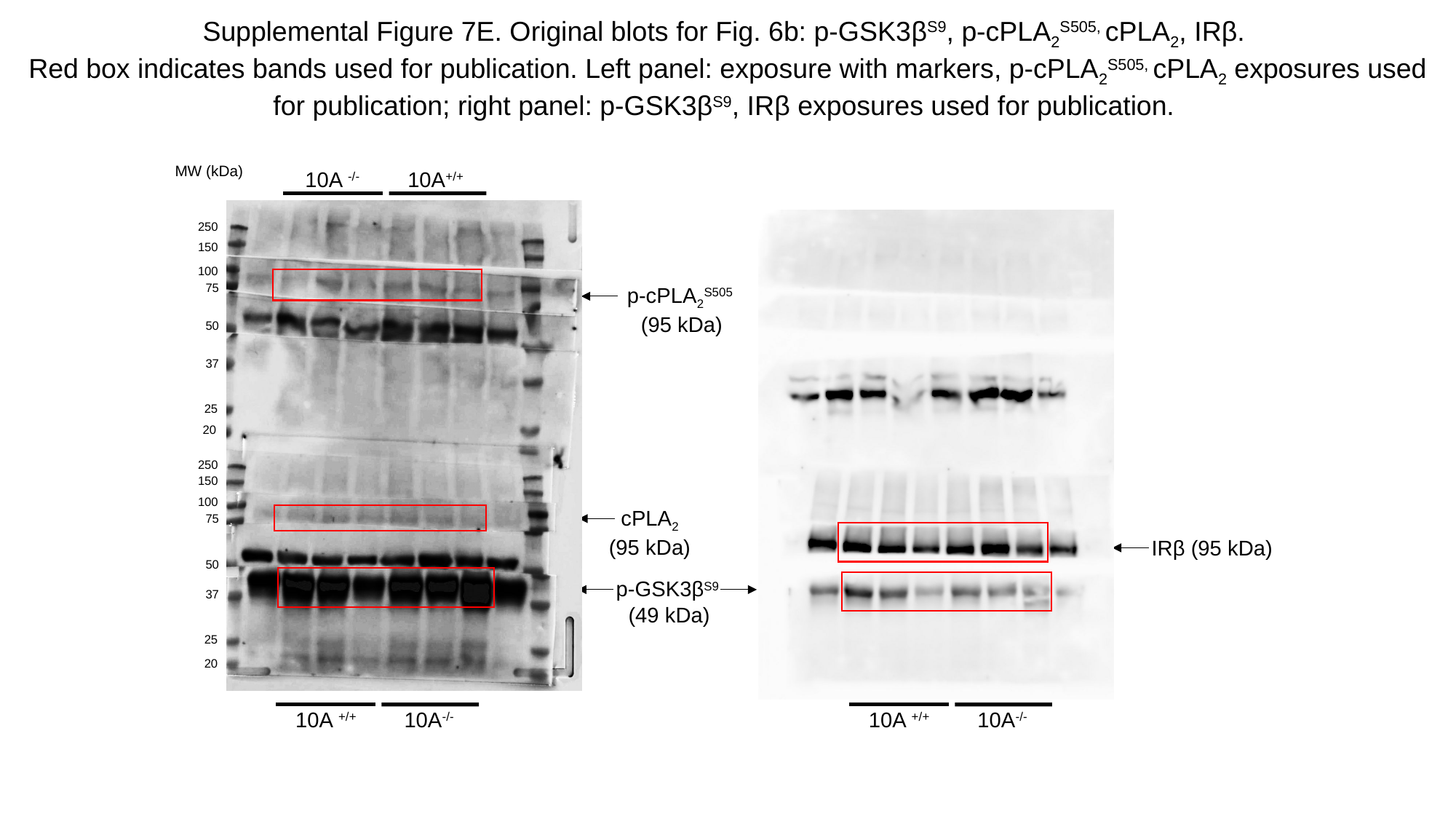

Supplemental Figure 7E. Original blots for Fig. 6b: p-GSK3βS9, p-cPLA2S505, cPLA2, IRβ.
Red box indicates bands used for publication. Left panel: exposure with markers, p-cPLA2S505, cPLA2 exposures used for publication; right panel: p-GSK3βS9, IRβ exposures used for publication.
MW (kDa)
10A -/- 10A+/+
250
150
100
75
p-cPLA2S505
(95 kDa)
50
37
25
20
250
150
100
cPLA2
(95 kDa)
75
IRβ (95 kDa)
50
p-GSK3βS9
(49 kDa)
37
25
20
10A +/+ 10A-/-
10A +/+ 10A-/-

## Slide 6
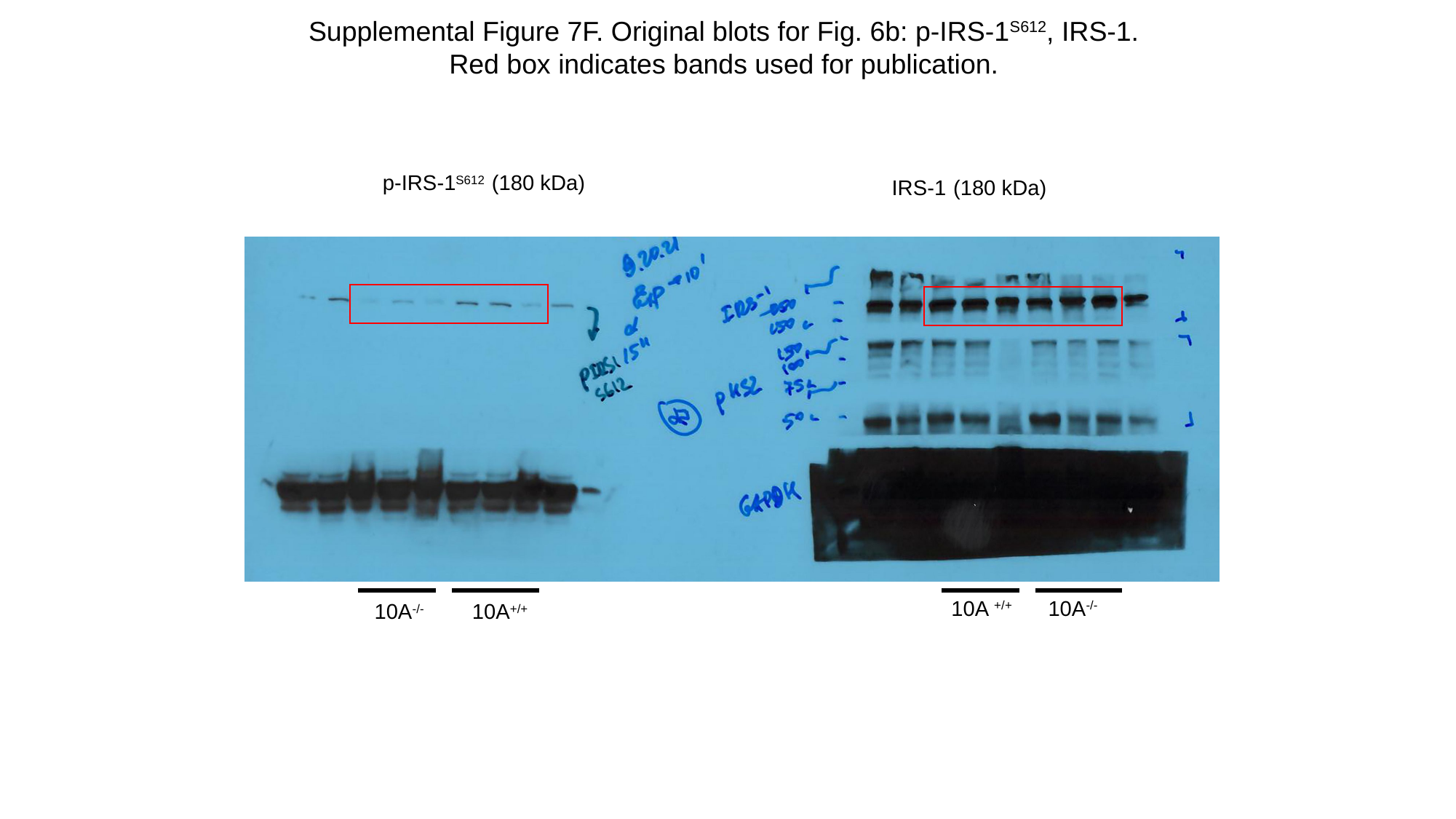

Supplemental Figure 7F. Original blots for Fig. 6b: p-IRS-1S612, IRS-1.
Red box indicates bands used for publication.
p-IRS-1S612 (180 kDa)
IRS-1 (180 kDa)
10A +/+ 10A-/-
10A-/- 10A+/+

## Slide 7
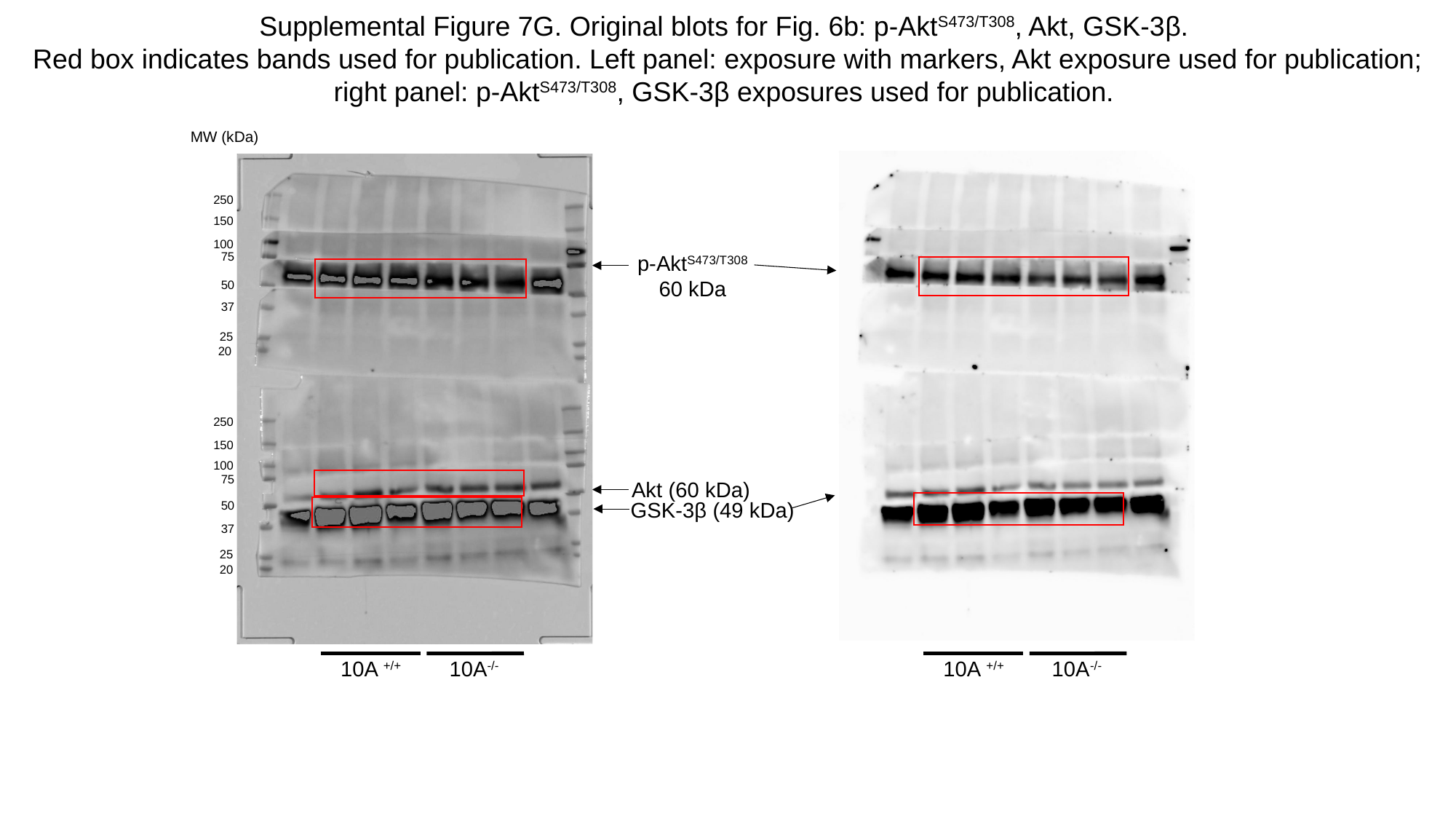

Supplemental Figure 7G. Original blots for Fig. 6b: p-AktS473/T308, Akt, GSK-3β.
Red box indicates bands used for publication. Left panel: exposure with markers, Akt exposure used for publication; right panel: p-AktS473/T308, GSK-3β exposures used for publication.
MW (kDa)
250
150
100
75
p-AktS473/T308
60 kDa
50
37
25
20
250
150
100
75
Akt (60 kDa)
GSK-3β (49 kDa)
50
37
25
20
10A +/+ 10A-/-
10A +/+ 10A-/-

## Slide 8
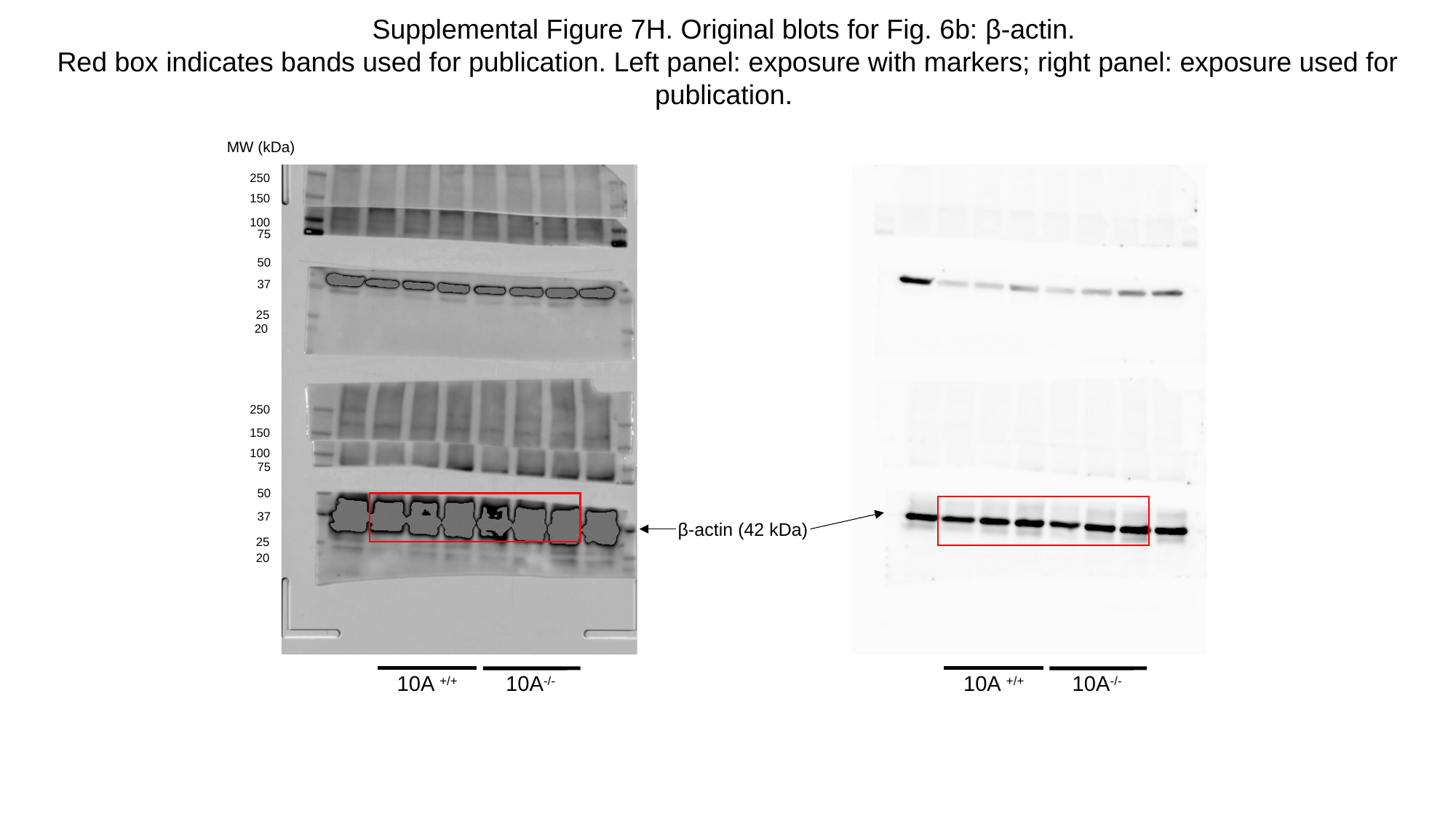

Supplemental Figure 7H. Original blots for Fig. 6b: β-actin.
Red box indicates bands used for publication. Left panel: exposure with markers; right panel: exposure used for publication.
MW (kDa)
250
150
100
75
50
37
25
20
250
150
100
75
50
37
β-actin (42 kDa)
25
20
10A +/+ 10A-/-
10A +/+ 10A-/-
